# Real-world integration of genomic data into the electronic health record: the PennChart Genomics Initiative

**DOI:** 10.1038/s41436-020-01056-y

**Published:** 2020-12-10

**Authors:** Kelsey S. Lau-Min, Stephanie Byers Asher, Jessica Chen, Susan M. Domchek, Michael Feldman, Steven Joffe, Jeffrey Landgraf, Virginia Speare, Lisa A. Varughese, Sony Tuteja, Christine VanZandbergen, Marylyn D. Ritchie, Katherine L. Nathanson

**Affiliations:** 1 Division of Hematology/Oncology, Department of Medicine, Perelman School of Medicine, University of Pennsylvania, Philadelphia, PA USA; 2 Division of Translational Medicine and Human Genetics, Department of Medicine, Perelman School of Medicine, University of Pennsylvania, Philadelphia, PA USA; 3 Information Services Applications, Penn Medicine, University of Pennsylvania, Philadelphia, PA USA; 4 Abramson Cancer Center, Perelman School of Medicine, University of Pennsylvania, Philadelphia, PA USA; 5 Department of Pathology and Laboratory Medicine, Perelman School of Medicine, University of Pennsylvania, Philadelphia, PA USA; 6 Department of Medical Ethics and Health Policy, Perelman School of Medicine, University of Pennsylvania, Philadelphia, PA USA; 7 Clinical Research, Ambry Genetics, Aliso Viejo, CA USA; 8 Department of Genetics, Perelman School of Medicine, University of Pennsylvania, Philadelphia, PA USA

Technologies in genomic medicine have rapidly evolved and transformed the ability to deliver precision medicine in nearly every field of health care. As genomic medicine has advanced, the electronic health record (EHR) has simultaneously been adopted into routine practice. A recent Points to Consider Statement by the American College of Medical Genetics and Genomics (ACMG) provides a framework for the optimal integration of genomic data into the EHR.^1^ The PennChart Genomics Initiative (PGI) at the University of Pennsylvania is a multidisciplinary collaborative effort including Penn Medicine clinicians, researchers, pathologists, legal staff, and information services with input and efforts from Epic Systems Corporation (Wisconsin) and Ambry Genetics Corporation (California), a commercial genetic testing laboratory. We describe our efforts to operationalize the ACMG guidelines in the “real world” to optimize our EHR (PennChart) for the delivery of precision medicine (Supplemental Fig. 1).

## INTEGRATION OF UNSTRUCTURED GENETIC DATA INTO THE EHR

We have taken a two-staged approach to integrating germline and somatic genetic data into the EHR. Currently, most genetic results are reported in unstructured PDF documents. We established common procedures across all Penn Medicine’s clinical genetics services for genetic testing reports, labeling them with a common naming convention and scanning them into a specific Genetic Results document type, which we created specifically for genetic testing results. This document filters both into our Lab (standard results) and PennChart Precision Medicine Tabs. We created the latter tab as a centralized location in the EHR to enable easy access to all genetic data, ensuring that it is not overlooked amid all the other testing that happens over a patient’s lifetime. This approach has standardized the real-time integration of unstructured genetic data into the EHR. Further, it has facilitated our efforts to import legacy data, as we began scanning all genetics documents with the common naming convention several years before implementing the Precision Medicine Tab.

## INTEGRATION OF DISCRETE GENETIC DATA INTO THE EHR

Although the ACMG recommends that genetic results be incorporated into patient records, at minimum, as scanned PDF files or images, it is preferable to store them in discrete, computable format to enable electronic searching, clinical decision support (CDS), and secondary use for research and operations.^2,3^ The second stage of our efforts therefore aimed to integrate structured genetic data into the EHR. The PGI has leveraged Epic’s Genomics Module to record discrete genetic variant information in Human Genome Variation Society (HGVS) nomenclature along with the notation of significance (e.g., *TP53* c.743G>A [p.Arg248Gln], pathogenic); transcript, genome build, chromosome, and genomic location are also included. Pharmacogenetic results are entered as diplotypes based on PharmVar star allele definitions (e.g., *DPYD* *1/*2A). Content experts throughout Penn Medicine collaborated to develop standard operating procedures (SOPs) to ensure institutional consistency for both manual and automated entry of discrete results into the Genomics Module. To date, these SOPs have been developed for autosomal dominant and pharmacogenetic variants with plans to expand to other result types over time, such as cytogenetics and autosomal recessive alleles. Manual entry of discrete genetic data into the Genomics Module is currently completed by genetics providers, who spend less than five minutes per variant.

Interfacing directly with genetic testing laboratories is essential to move from manual to automated entry of discrete results into the EHR. The PGI partnered with Ambry Genetics to implement computerized order entry so providers can place genetic test orders directly into PennChart for import into the Ambry portal. The return of results has followed a phased approach using a Health Level 7 (HL7) interface, first with PDF reports and then with discrete results, which are automatically imported into the Genomics Module, linked to their associated PDF documents, and accompanied by direct provider notification. We are working on expanding our HL7 capacity with other commercial genetic testing laboratories.

The ACMG also recommends that updated genetic test results be clearly linked to the original report in the EHR, as the interpretation of results may evolve and result in reclassifications over time.^2^ The PGI’s partnership with Ambry Genetics enables the automatic import of results both at the time of initial testing and as variant reclassification occurs. If an update occurs, a notification is sent to the ordering provider and care team for review of the amended report.

## LINKAGE OF GENETIC DATA TO CLINICAL DECISION SUPPORT

One of the greatest potential benefits of integrating genetic data into the EHR is the ability to link results to CDS.^2,4^ Not only should providers be able to retrieve external educational content to learn more about a patient’s genetic findings, but they should also receive automated recommendations at the point of care to facilitate clinical decision-making.^5,6^ The ACMG highlights that EHR vendors may not be fully equipped to build CDS systems in isolation due to the complex and dynamic nature of genomic medicine. The multidisciplinary nature of the PGI addresses this challenge by fostering collaboration between clinical, pathology, and information technology (IT) experts to build CDS tools that are seamlessly implemented into routine care.

The PGI has leveraged Epic’s Genomic Indicators, driven by variants that are pathogenic/likely pathogenic or medically actionable, which are tags added to a patient’s record to indicate potential disease risk or drug sensitivity based on his/her genetic testing results. Genomic indicators are displayed on the Snapshot Tab (chart front page) and facilitate clinical decision-making by triggering automated recommendations directly in the EHR targeted to both providers and patients. By using triggered genomic indicators, we prevent variants of uncertain significance from being misinterpreted by nongenetics providers as disease associated. Our initial use cases for CDS provide guideline-concordant recommendations on colonoscopy timing intervals for patients with Lynch syndrome and fluoropyrimidine dose adjustments in patients with dihydropyrimidine dehydrogenase deficiency identified on *DPYD* gene testing.^7,8^

## PATIENT ACCESS TO GENETIC DATA

The ACMG recommends that genetic data in the EHR be made available to patients in understandable and usable form. At the time of pretest counseling, patients are informed that they will receive their genetic results in conjunction with counseling on their clinical interpretation, thereby mitigating any confusion and distress that may arise if patients receive their results in isolation. As such, we have developed a method to retain these data, as permitted by law, until patients are counseled on their results, after which ordering genetics providers manually release them concurrently to nongenetics providers and through PennChart’s secure patient portal. This electronic portal also features the Genetic Profile, a centralized location where patients may view their results, along with annotated educational information written in patient-friendly language for select genetic conditions and pharmacogenetic results.

## SECURITY AND PRIVACY PROTECTIONS

Although our primary objective in developing the Genetic Results document type was to enable centralized document display in the Precision Medicine Tab to improve patient care, it also provides the flexibility to segregate genetic results from other EHR data. Being able to segregate genetic data was found to be important by the privacy officer at our institution to provide the capability to withhold that data when desired for privacy purposes, such as in certain health information exchange use cases. Genetic results from family member records also are categorized as a distinct document type within the EHR so that they are only accessible to genetics providers and cannot be released to external parties as part of the patient’s chart.

## CONCLUSIONS

The PGI has made significant strides in integrating genomic data into the EHR for the optimization of patient care. To date, approximately 21,500 documents have been filtered into the Precision Medicine Tab, including over 3,500 legacy scans and 627 discrete results from Ambry Genetics. We attribute our successes to date to large-scale, collaborative, and coordinated efforts led by committed champions with clinical, research, pathology, IT, and legal expertise throughout Penn Medicine. However, our experiences have not been without challenges. The complexity of genomic medicine requires substantial educational initiatives to ensure that our multidisciplinary team has the knowledge base necessary to effectively impact patient care. In addition, as we expand our discrete reporting interfaces to other laboratories, they, too, must have the infrastructure necessary to connect with our EHR systems. Finally, the PGI has benefited from significant support and investment from Penn Medicine that may not be feasible at other, smaller institutions. As such, we are committed to sharing our SOPs, decision process algorithms, and other documentation with the genomic medicine community to inform broader efforts to optimize the integration of genomic data into the EHR.

Future PGI endeavors include (1) developing SOPs beyond autosomal dominant and pharmacogenetic variants for other types of genetic variants, (2) building CDS systems for additional genetic test results, (3) providing guidance on genetic testing indications and familial risk assessment, (4) partnering with additional genetic testing laboratories for computerized order entry and discrete result reporting, (5) collaborating with other health-care facilities to enable transportability of genetic data for patients seeking care at multiple institutions, and (6) maintaining privacy and security protections as genetic data become more accessible electronically, both within and between institutions. We also look forward to working with the genomic medicine community to develop innovative solutions for broader-reaching goals such as unifying reporting standards, storing high-volume genomic data efficiently, supporting variant reclassifications from external knowledge bases, handling genetic results from unique populations, and ensuring equitable access to genomic medicine for all patients. The EHR is a powerful tool for the delivery of precision medicine; we are optimistic that the efforts of the PGI and other similar initiatives will be instrumental in optimizing patient care and accelerating the discovery of novel genomic medicine applications over time.

## Supplementary information

Supplemental Figure 1
