# Supplementary material for: Real-world integration of genomic data into the electronic health record: the PennChart Genomics Initiative
Source: Genet Med. 2020 Dec 10;23(4):603–5. doi: 10.1038/s41436-020-01056-y (PMC8026392; doi:10.1038/s41436-020-01056-y)
Supplement: Supplementary file 1 — Supplemental Figure 1 [file 41436_2020_1056_MOESM1_ESM.pdf]

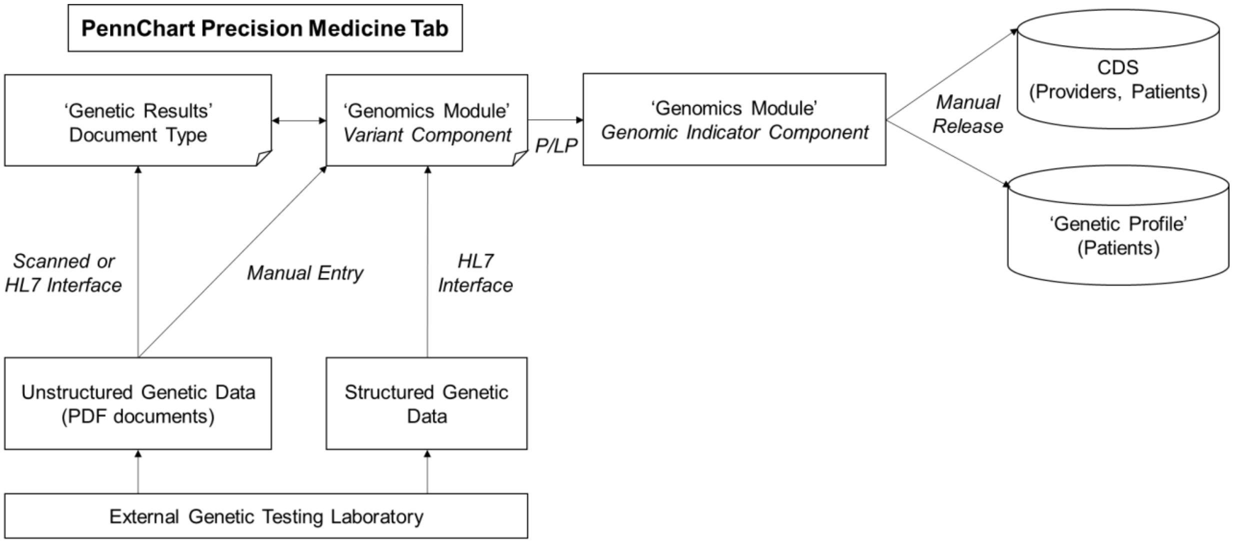

Supplemental Figure 1. Integration of genomic data into PennChart, the electronic health record system at Penn Medicine. HL7 = Health Level 7. P/LP = pathogenic/likely pathogenic variant. CDS = clinical decision support.
